# Trends and Impact Factors of Mental Health Service Utilization among Resettled Humanitarian Migrants in Australia: Findings from the BNLA Cohort Study

**DOI:** 10.3390/ijerph191610119

**Published:** 2022-08-16

**Authors:** Meng Zheng, Feng Chen, Yan Pan, Di Kong, Andre M. N. Renzaho, Berhe W. Sahle, Rashidul Alam Mahumud, Li Ling, Wen Chen

**Affiliations:** 1 Department of Medical Statistics, School of Public Health, Sun Yat-sen University, Zhongshan Road 2, Guangzhou 510080, China; 2 Center for Migrant Health Policy, Sun Yat-sen University, Zhongshan Road 2, Guangzhou 510080, China; 3 School of Medicine, Translational Health Research Institute, Western Sydney University, Campbelltown, NSW 2751, Australia; 4 Maternal, Child and Adolescent Health Program, Burnet Institute, Melbourne, VIC 3004, Australia; 5 School of Nursing and Midwifery, Faculty of Health, Deakin University, Melbourne, VIC 3125, Australia; 6 Centre for Quality and Patient Safety Research, Alfred Health Partnership, Institute for Health Transformation, Melbourne, VIC 3125, Australia; 7 Melbourne School of Population and Global Health, University of Melbourne, Melbourne, VIC 3125, Australia; 8 NHMRC Clinical Trials Centre Faculty of Medicine and Health, The University of Sydney, Camperdown, NSW 2006, Australia; 9 Centre for Health Research, University of Southern Queensland, Toowoomba, QLD 4350, Australia

**Keywords:** humanitarian migrants, mental health service, impact factors, resettlement, gender difference, BNLA project

## Abstract

Resettled humanitarian migrants (HMs) have high levels of mental disorders, but factors associated with the utilization of mental health services (MHS) are poorly understood. We aimed to explore trends and impact factors of MHS utilization among HMs in the process of resettlement in Australia. A total of 2311 HMs from the 1st (2013), 3rd, and 5th (2018) waves of a national cohort study were included. MHS utilization in the past year was assessed by two indicators: having MHS contacts and the frequency of MHS contacts. Trends were identified by Cochran–Armitage tests, and generalized linear mixed models and ordered logistic models were fitted to explore impact factors of MHS utilization. The proportion of having MHS contacts significantly rose from 13.0% to 29.4% over the five years. MHS utilization was mainly driven by perceived needs, such as post-traumatic stress disorders and the degree of post-migration stress. Unemployment and strong belongingness to the local community were also associated with having MHS contacts. No significant gender difference was found in having MHS contacts but females tended to contact MHS more frequently. Resettled HMs have a persistent dilemma of high mental illness prevalence and MHS underutilization. Sustainable mental health education and long-term resettlement services targeted at social integration that consider gender difference are urgently needed in host countries.

## 1. Introduction

According to the United Nations High Commissioner for Refugees, 82.4 million people worldwide were forcibly displaced in 2020, of whom 30.5 million were refugees and asylum seekers, hereinafter called humanitarian migrants [1]. There are three pillars promoted by the international community to conceptualize the so-called ‘durable solutions’ framework to enable humanitarian migrants to live a dignified life: voluntary repatriation (encouraging refugees to return home), local integration (integrating refugees within the host communities), and resettlement in a third country [2]. Australia was 1 of the only 29 countries around the world accepting UNHCR submissions in 2020, resettling 7048 refugees (around 8.6% of the worldwide resettled humanitarian migrants) in 2019 [3,4]. Newly resettled humanitarian migrants in Australia have been reported to have serious mental health problems, yet they underutilize mental health services (MHS) [5,6,7]. 

As suggested by existing work, when seeking for MHS, humanitarian migrants may face barriers, such as stigma and lack of knowledge, financial hardships, limited language proficiency, transport difficulties, and housing instability, as well as barriers related to individual experience, such as immigration status [8,9,10,11]. In addition, demographic characteristics, including age, gender, education level, and health status, have been considered to impact MHS utilization [12,13,14,15]. However, current evidence on associations between these factors (such as gender and age) and MHS utilization remains inconsistent [16,17,18]. One possible explanation of these inconsistent findings could be that most studies on MHS utilization among humanitarian migrants were qualitative or cross-sectional with limited sample sizes, and they were conducted at different periods of resettlement. More importantly, previous literacy seldom uses theoretical frameworks to delineate factors to be examined. Therefore, longitudinal studies that cover a continuous process of resettlement and theoretical framework are needed to confirm predictors of MHS utilization, which are critical to identify opportunity for early intervention to bridge the treatment gap.

The existing literature widely adopted theories, including the theory of planned behavior, the social ecological model, and Andersen’s behavioral model of health service use (hereinafter called Anderson’s framework), when exploring impact factors of healthcare utilization [19,20,21]. In which, Anderson’s framework is one of the most widely used frameworks that present dynamic and recursive relationships between contextual characteristics, individual characteristics, and health service utilization [22]. The model identifies three clusters of individual characteristics, namely predisposing characteristics, enabling resources, and perceived needs [19,23]. Predisposing characteristics include variables, such as age, gender, education level, and health beliefs. Enabling resources include variables, such as income and living arrangements, as well as service access issues, such as transportation difficulties, language, or financial hardships. Perceived needs mainly cover how people view their general health and functional state, and their judgment of whether or not to seek medical help [24,25]. The application of Andersen’s framework using data from longitudinal studies fills in an important gap.

Additionally, existing research only assessed the access to MHS utilization among humanitarian migrants, the frequency of utilization was ignored [26]. Regular MHS utilization has been confirmed as a key impact factor of the treatment effectiveness and mental health outcomes, but it might be poor in patients of mental disorders [27]. Understanding the frequency of MHS utilization and its correlates will promote the effectiveness of MHS and mental health in humanitarian migrants.

Guided by Andersen’s framework, the current study used a representative cohort to explore (1) the trends of MHS utilization among humanitarian migrants in five years of resettlement, and (2) impact factors of MHS utilization both in perspectives of accessibility and frequency. The findings from this study will inform targeted resettlement services to promote MHS utilization in the most vulnerable population, thus making a significant contribution to the SDG target 3.4, which seeks to promote mental health and well-being [28].

## 2. Materials and Methods

### 2.1. Data Source

This study used data from the Building a New Life in Australia (BNLA) project, a national cohort of 2399 newly resettled humanitarian migrants in Australia, which followed the participants over five years, from October 2013 to February 2018 [29]. To accommodate different cultural backgrounds, all survey materials were translated into 19 different languages. Face-to-face interviews were conducted at the 1st, 3rd, and 5th waves using computer-assisted self-interviews, computer-assisted personal interviews, and interviews with the help of interpreters. Telephone interviews were adopted at the 2nd and 4th waves.

### 2.2. Participants and Sampling

The BNLA project included humanitarian migrants whose permanent visas (offshore or onshore visas) were granted (start of resettlement) within 3–6 months before the 1st wave. A multistage sampling process was adopted. First, 11 sites across Australia were selected as study settings because a large number of humanitarian migrants were set there between November 2010 and October 2011 [30]. Second, 4035 eligible migrating units (groups of migrants using the same migration application) in the 11 sites were identified with two inclusion criteria: (1) having been granted a permanent visa between May and December 2013; (2) the principal applicant (PA) in the migrant unit aged 18 years or older. Third, Colmar Brunton Social Research interviewers contacted PAs in eligible migrant units to involve them in the study. If the PA agreed to participate, secondary applicants (SAs) aged over 14 years in the same migrant units were invited to participate voluntarily. The BNLA participants were consisted of PAs and SAs. Baseline information was collected in 2013 (the 1st wave), and 4 annual subsequent waves were conducted between 2014 to 2018 (the 2nd to 5th wave).

**Figure 1:**
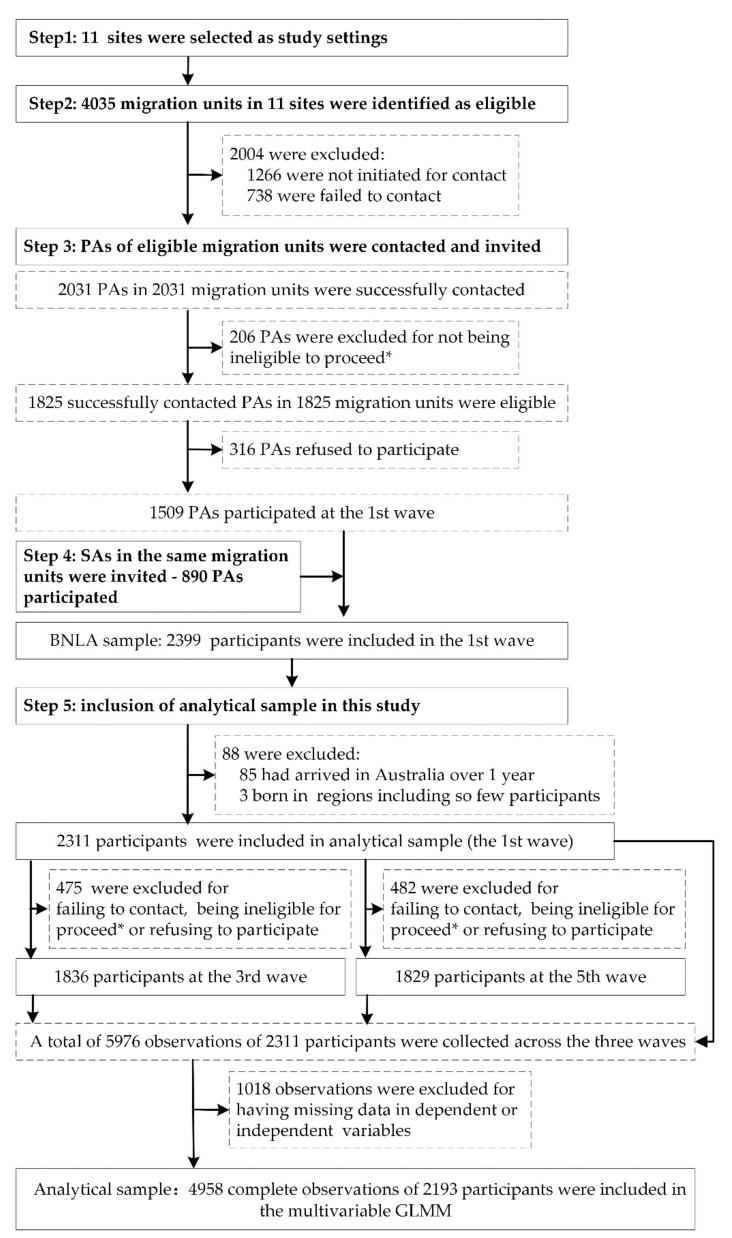
Flow chart of the sampling. Note: context in solid boxes are sampling steps and sampling outcomes at each step, where sampling steps are bold; context in dashed boxes are detailed operation of each step. * Ineligible for proceed: successfully contacted but could not proceed with an interview for reasons, such as the quota was met, moved to an area outside the scope of interviewing, or unavailable for the duration of the fieldwork period. GLMM: generalized linear mixed model, PAs: principal applications, SAs: secondary applicants.

Our analytical sample (Figure 1) only included the BNLA participants who had arrived in Australia within one year at the first wave. Observations in the 2nd and 4th waves were excluded due to some important variables of interest, such as having MHS contacts and post-migration stress, not being collected in secondary applicants by shortened questionnaires administered by telephone interviews.

### 2.3. Measurements

#### 2.3.1. Outcome Variables: MHS Utilization

The primary outcome was MHS utilization, defined as contacts with any type of mental health services for emotional problems provided by professional workers (including doctors, counselors, and psychologists). Two self-reported indicators were used to assess MHS utilization: having MHS contacts and the frequency of MHS contacts. Having MHS contacts was a binary variable to identify whether used MHS or not in the past year. Participants were asked “Have you been given any medications by a doctor for emotional problems since you arrived in Australia?” at the 1st wave (0 = no; 1 = yes). In follow-up waves, “In the last 12 months, have you received help from a professional, such as a doctor, counselor, or psychologist to help you deal with emotional problems?” was asked. Some participants had arrived in Australia for more than one year at the start of the project. To keep consistent in measurements at different waves, we only included participants who had arrived within one year at the 1st wave. The frequency of MHS contacts was investigated at 3rd and 5th waves. Participants who had MHS contacts were further asked “How often have you received help for emotional problems in the last 12 months?”. Four choices were provided in the original questionnaire, including 1–2, 3–6, 6–9, and ≥10 times.

#### 2.3.2. Explanatory Variables

We chose explanatory variables based on Andersen’s framework, which concluded explanatory variables as predisposing characteristics, enabling resources, and perceived needs (see Supplementary Material Figure S1). The way of constructing all variables and their explanation are in the appendix (see Supplementary Material Table S1).

Predisposing characteristics included age, gender, region of birth, and education level. Region of birth of the analytical sample was classified into four in terms of the Standard Australia Classification of Country (SACC-major) [31]. Enabling resources included employment status, financial hardships, belongingness to the Australian community, housing arrangement, and transport difficulty. Perceived needs included the degree of post-migration stress, as well as indicators suggesting physical and mental health status, which included overall health, post-traumatic stress disorder (PTSD), and high risk of severe mental illness (HR-SMI). The degree of post-migration stress was a count variable (0–10) that derived from whether the participants perceived ten types of post-migration stressors. Higher number of stressors suggests higher degree of stress. 

PTSD symptoms in the past week was assessed by the PTSD-8, which is a standardized cross-cultural eight-item instrument derived from the Harvard Trauma Questionnaire Part IV [32]. The PTSD-8 is validated against the *Diagnostic and Statistical Manual of Mental Health Conditions, Fourth Edition* and covers all three core groups of PTSD symptoms: intrusion, avoidance, and hyper-arousal [33]. Each item was answered on a four-point Likert-type scale (1 = not at all, 4 = most of the time). At least one item scored 3–4 in each group indicates the presence of PTSD. The internal consistency of the PTSD-8 in the BNLA project is 0.96 [34].

Kessler Screening Scale for Non-specific Psychological Distress (K6) was used to identify HR-SMI. With limited resources for treatment, people with mental health conditions are distinguished not only based on diagnosis but severity to define medical necessity. K6 is one of the most used scales to identify people with relatively severe non-specific psychological distress that need medical care. Participants screened positive were called people with HR-SMI [35]. Participants were asked six questions about how often they had six kinds of feelings in the past four weeks, and the answer for each question ranged from 1 (none of the time) to 5 (all of the time). The summed score ranged from 6–30, and 19 or higher scores mean having a HR-SMI [31]. The internal consistency of the K6 in the BNLA project was 0.93 [34].

### 2.4. Statistical Analysis

Statistical analyses were conducted using R (version 4.1.1) and STATA (version 16.0). Mean and standard deviation (SD) were computed for continuous variables, while frequency and proportion were reported for categorical variables. MHS utilization, PTSD, and HR-SMI were described both in overall and gender-group samples, and gender differences at each time were identified by chi-square tests. Cochran–Armitage tests were used to test unadjusted time trends for categorical variables and generalized linear mixed models (GLMMs) for continuous variables.

To explore long-term impact factors of having MHS contacts, we established GLMMs using data from the 1st, 3rd, and 5th waves. Bivariate and multivariable models were established to explore unadjusted and adjusted effects. Odds ratios (OR) and their 95% CIs were calculated to assess the effects accordingly. For each model, resettlement time was included as a fixed effect to account for changes in MHS utilization over time, and individual was set as a random intercept to account for repeated measures. 

Considering there was no significant difference in the frequency of MHS contacts between the 3rd and 5th waves, we took data from participants who had MHS contacts at the latest wave (the 5th wave) to investigate factors associated with the frequency of MHS contacts. We conducted chi-square tests to select potential influential factors (see Supplementary Material Table S2). As the frequency of contacts is collected as an ordered variable, bivariate and multivariable ordered logistic regression models were fit and OR and their 95% CIs were calculated.

Missing rate were low across all variables (under 5% except for the degree of post-migration stress, which is 6.5%). We excluded observations with missing data from the GLMMs. Besides, population weights were used to adjust for non-response in the multivariable GLMM, and a sensitivity analysis was conducted. In the ordered logistic regression model, multiple imputations were used for missing data on independent variables (missing rates: 0.8–3.8%), and a sensitivity analysis was conducted to identify how robust our results are.

## 3. Results

### 3.1. Characteristics of Study Participants

A total of 2311 participants were included in this study, among them, 1836 (retention rate: 79.4%) and 1829 (retention rate: 79.1%) were successfully followed up at the 3rd and 5th waves, respectively (Figure 1). The mean age was 35.50 (SD = 14.01) years with 53.9% males. Participants were predominantly from North Africa and the Middle East (55.1%) followed by South and Central Asia (34.5%). Workforce participation increased from 5.4% to 29.9% whilst there was a decrease in the proportion of humanitarian migrants reporting facing financial hardships (from 41.8% to 33.7%) and hardly ever or never had belongingness to the Australian community (from 8.0% to 5.4%) (Table 1).

### 3.2. Trends of the Prevalence of Common Mental Health Conditions among Resettled Humanitarian Migrants

**Figure 2:**
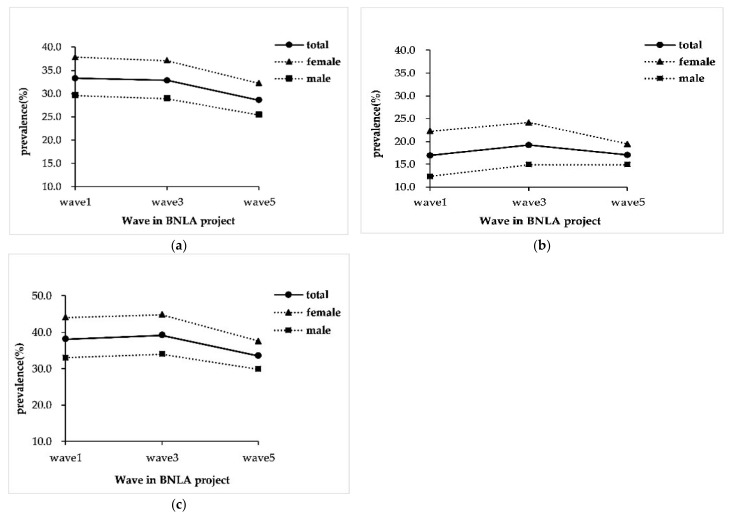
Trends of the prevalence of common mental health conditions among resettled humanitarian migrants in Australia. Note: (**a**) The prevalence of post-traumatic stress disorder (PTSD) in the five years of resettlement; (**b**) The prevalence of high risk of severe mental illness (HR-SMI); (**c**) The prevalence of PTSD and/or HR-SMI. The difference between females and males was tested by chi-square test at each wave, with all *p* < 0.05.

Mental health conditions were prevalent in humanitarian migrants. The prevalence of PTSD was 33.3%, 32.8%, and 28.6% at the 1st, 3rd, and 5th wave, respectively, with a decreasing trend (*p* < 0.001). However, HR-SMI maintained a high prevalence (16.9%, 19.2%, and 17.0%, *p* = 0.953). Considering comorbidity, the prevalence of PTSD and/or HR-SMI were displaying a significant reduction in total participants (38.4%, 39.2%, and 33.7%, *p* = 0.004) but maintained at a high level among males (33.0%, 34.0%, and 29.9%, *p* = 0.147). Female humanitarian migrants suffered more from mental health conditions than males, and this remained consistent over time (see Figure 2; Supplementary Material Table S3).

### 3.3. Trends of the MHS Utilization among Resettled Humanitarian Migrants

**Figure 3:**
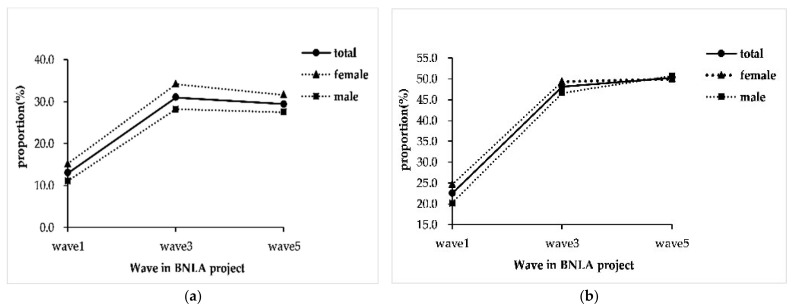
The trends of MHS utilization among resettled humanitarian migrants in Australia. Note: (**a**) The proportions of having MHS contacts in the overall participants; (**b**) The proportions of having MHS contacts in the participants with post-traumatic stress disorder (PTSD) and/or high risk of severe mental illness (HR-SMI). The difference between females and males was tested by chi-square test at each wave, with all *p* < 0.05 in (**a**) and all *p* > 0.05 in (**b**). MHS: mental health service.

The proportion of humanitarian migrants who had MHS contacts increased with resettlement time (Figure 3), from 13.0% (298/2311) at the 1^st^ wave, to 31.0% (556/1836) at the 3^rd^ wave and 29.4% (530/1829) at the 5^th^ wave (*p* < 0.001). The crude proportion of having MHS contacts in females were 15.2%, 34.2%, and 31.6% at the three waves, which were higher than those in males (11.1%, 28.2%, 27.5%; *p*_1_ = 0.004, *p*_3_ = 0.007, *p*_5_ = 0.052). In participants with PTSD or/and HR-SMI, the proportion of having MHS contacts were 22.5%, 48.1%, and 50.3%, respectively. There was no significant difference between females (24.6%, 49.3%, 50.0%) and males (20.1%, 46.6%, 50.7%, *p*_1_ = 0.117, *p*_3_ = 0.488, *p*_5_ = 0.860).

The frequency of MHS contacts did not vary during 3.5–5.5 years of resettlement (*p* = 0.360). Less than 50% of participants who came into MHS contacts used them six or more times in a year, but this varied by gender. Nearly 50% (127/252) of females contacted MHS six or more times a year, compared to 38.9% (96/247) for males (*p* = 0.017). In participants with PTSD and/or HR-SMI, 60.3% (91/151) of females used MHS more than six times a year, whilst 47.4% (64/135) for males (*p* = 0.029).

### 3.4. Impact Factors of Having MHS Contacts among Humanitarian Migrants

A total of 4958 complete observations were included in the multivariable GLMM (Table 2). All indicators in perceived needs had significant impacts on MHS contacts. Over time, MHS contacts were more common among humanitarian migrants reporting higher degree of post-migration stress (aOR = 1.06, 95% CI: 1.01–1.11), poor overall health (aOR = 1.41, 95% CI: 1.31–1.52), PTSD (aOR = 2.08, 95% CI: 1.72–2.52), and HR-SMI (aOR = 1.99, 95% CI: 1.59–2.49). Meanwhile, some enabling resources indicators showed significant long-term impacts on the MHS contacts. The probability of having MHS contacts was 29% lower among humanitarian migrants who were employed (aOR = 0.71, 95% CI 0.55–0.92) and 51% higher among those reporting strong belongingness to the Australian community (aOR = 1.51, 95% CI: 1.03–2.21). In predisposing characteristics, a higher probability of having MHS contacts was associated with older age (aOR = 1.16, 95% CI: 1.04–1.28). Notably, there was no significant gender difference after controlling other variables (aOR = 0.99, 95% CI: 0.82–1.19). The results remained robust in the analysis, based on weighted data (see Supplementary Material Table S4).

### 3.5. Factors Associated with the Frequency of MHS Contacts among Humanitarian Migrants

A total of 499 participants reported the frequency of MHS contacts at the 5th wave (see Supplementary Material Table S2). The ordered logistic model showed predisposing characteristics (gender and seven or more years of schooling (aOR = 1.57, 95% CI: 1.07–2.30)) and perceived needs (overall health (aOR = 1.45, 95% CI: 1.22–1.71) and PTSD (aOR = 1.93, 95% CI: 1.33–2.82)) were significantly associated with the frequency of MHS contacts, but HR-SMI showed no significant association (Table 3). Notably, female humanitarian migrants used MHS more often than males after controlling for other variables (aOR = 1.54, 95% CI: 1.05–2.26). Significant variables and their aOR values were similar when using complete or imputed data (see Supplementary Materials Table S5), suggesting that our results were robust.

## 4. Discussion

Our study adds to the limited evidence of the trends and impact factors of MHS utilization among resettled humanitarian migrants. We found a persistent treatment gap of mental health conditions. Then, based on Andersen’s framework, we found that a higher possibility of having MHS contacts was associated with more perceived needs, more financial hardships, unemployment, and older age throughout the five years of resettlement. To our knowledge, this is also one of the first studies to explore influential factors of the frequency of MHS utilization among humanitarian migrants, which found females used MHS more frequently. Altogether, our study provides evidence for sustainable targeted services for resettled humanitarian migrants to promote MHS utilization, which will contribute to SDG’s goal to reduce mental health illness and promote mental health and wellbeing.

In line with other research, we found a significantly higher prevalence of PTSD and HR-SMI in female humanitarian migrants [7,15]. Although there were more female humanitarian migrants having MHS contacts than males, no significant gender difference existed after controlling for mental health conditions [16]. It means that the gender difference in MHS contacts among humanitarian migrants could be fully explained by a higher prevalence of mental problems in females. Moreover, our findings suggest an obvious treatment gap in humanitarian migrants [9,36]. Although the persistently high prevalence of mental health conditions (38.4%, 39.2%, and 33.7%), there were lower proportions that had access to MHS (13.0%, 31.0%, and 29.4%), leaving nearly half of resettled humanitarian migrants with mental health symptoms that had not been diagnosed or treated independently of gender throughout nearly 5 years of resettlement. In contrast, there is a higher proportion of accessing (17%) than that of people in need for MHS (15%, screened by Kessler Screening Scale) in Australian general population [37]. Therefore, sustainable measures to promote MHS contacts are urgently needed in the long-term process of resettlement.

As a minimally adequate treatment consists of receiving eight or more times according to evidence-based guidelines [38], the frequency of MHS contacts kept at a low level throughout the resettlement process means the inadequate use of MHS utilization in humanitarian migrants. However, notably, we found that female humanitarian migrants were likely to use MHS more often after adjusting for health status and other covariates. This might be because male humanitarian migrants are used to tolerating discomfort after social learning to express fewer needs for MHS than females [13,39]. Besides, it may be hard for males to use MHS quite often for fear of losing their job, power, and pride [40]. These findings suggested that the frequency of MHS utilization is needed to be improved through measures, such as strengthened clinical follow-up and thematic health education, especially for male humanitarian migrants. 

As suggested by Andersen’s framework, MHS utilization among humanitarian migrants would primarily be driven by perceived needs [41,42]. The proportion of having MHS contacts in humanitarian migrants with mental health conditions (approximately 50%) was much higher than that in the overall participants (approximately 30%), indicating that perceived symptoms play dominant roles in promoting contacts for MHS among humanitarian migrants in Australia. However, the fact that nearly half or even more [36] of humanitarian migrants with mental health symptoms had no MHS contact and that HR-SMI was not related to frequency of MHS utilization, suggests that the symptoms that need MHS might not be adequately perceived [10,43], especially in humanitarian migrants with HR-SMI. A higher degree of post-migration stress might promote MHS contacts through producing more perceived needs, which could be explained by the associations between post-migration stress and health status. The post-migration stress humanitarian migrants faced is mainly related to social integration and loneliness, and they are proved to be positively associated with PTSD, HR-SMI, and poorer overall health directly [15,30,44]. Furthermore, they could mediate the impact of pre-migration trauma on PTSD [34]. Meanwhile, stronger belongingness to the Australian community, which is critical for cultivating social integration [45], is positively associated with MHS contacts. Thus, health education will help to meet existing needs for MHS, and resettled services aimed at addressing post-migration stress, especially social integration problems, will help reduce needs in future fundamentally. 

To our surprise, employed humanitarian migrants had a lower possibility of MHS contacts, since those with paid work may have more financial resources to access health care [8,46]. One explanation is that employed HMs may have better health conditions because health is important human capital [47]. Another explanation might be that they lack accessibility to MHS for following reasons. Utilizing MHS will cause more indirect costs because of missed workdays. What is more, it is worth noting that although the employment rate increased in the five years, the proportion of participants who were employed was still low (29.9%), indicating the persisting difficulty in finding a paid job for humanitarian migrants. Humanitarian migrants may especially fear the termination of hard-won jobs because of experiencing obvious symptoms or diagnosis of mental health conditions, thus they are reluctant to use MHS [48]. Our findings suggest that services addressing unemployment and stigma of mental health conditions in the work environment are needed to facilitate plausible MHS utilization. 

There are several limitations of this study. First, although the data on measurements were obtained using adopted standardized and validated methods in terms of cultural and social contexts, the main sources of bias were self-report, instrument bias (e.g., one item to measure MHS utilization). Especially, the measurement of having MHS contacts was different at the 1st wave, which asked for MHS utilization since arriving in Australia and the duration varied from 3 months to 3 years. We excluded participants living for more than one year to keep consistent in measurement. Although most of the people in the analytical sample still arrived in Australia less than 12 months prior, which will cause an underestimation of utilization, we think that having MHS contacts would been less influenced as a binary indicator. Second, although a comprehensive consideration of the potential factors was conducted according to Anderson’s framework, some factors, such as the stigma of illness [49], might be important barriers for MHS utilization but were not measured in the BNLA datasets. Third, we adopted a cross-sectional analysis when exploring factors associated with the frequency of MHS contacts, so that causal associations cannot be established. Finally, although we found significant gender differences in the frequency of MHS utilization, we did not conduct analyses stratified by gender on account of the limited sample size. It informs that further prospective designs are needed to explore gender difference in MHS utilization.

## 5. Conclusions

Humanitarian migrants in Australia continued to underutilize MHS throughout the first five years of resettlement independent of gender, while there was less frequency of utilization in males. Perceived needs, including perceived mental symptoms and post-migration stress and strong belongingness to the Australian community, promoted MHS utilization. Sustainable mental health education and resettlement services targeted at post-migration stress that consider gender difference are needed to deliver equitable and universal mental health care in the most vulnerable population, which may promote mental health under the SDG target 3.4. More studies on these impact factors are needed to close the treatment gap.

## Figures and Tables

**Table 1:** Individual characteristics and mental health service utilization of BNLA humanitarian migrants at three waves.

Variables	Wave 1 (*n* = 2311)	Wave 3 (*n* = 1836)	Wave 5 (*n* = 1829)	*p*
**Outcomes**				
Having MHS contacts, No. (%)	298 (13.0)	556 (31.0)	530 (29.4)	<0.001
Frequency of MHS contacts, No. (%)				0.360 ^1^
1–2 times	NA	160 (29.6)	119 (23.8)	
3–5 times	NA	131 (24.2)	157 (31.5)	
6–9 times	NA	97 (17.9)	82 (16.4)	
≥10 times	NA	153 (28.3)	141 (28.3)	
**Predisposing characteristics**				
Age, mean (SD), year	35.5 (14.0)	36.2 (14.3)	36.1 (14.2)	NA
Gender (female), No. (%)	1065 (46.1)	869 (47.3)	867 (47.4)	NA
Region of birth, No. (%)				
North Africa and the Middle East	1286 (55.7)	1036 (56.4)	1079 (59.0)	NA
South-East Asia	134 (5.8)	104 (5.7)	98 (5.4)	NA
South and Central Asia	798 (34.5)	641 (34.9)	597 (32.6)	NA
Sub-Saharan Africa	93 (4.0)	55 (3.0)	55 (3.0)	NA
Education level, No. (%)				
Never attend school	376 (16.4)	298 (16.4)	287 (15.8)	NA
≤6 years of schooling	470 (20.5)	398 (21.9)	390 (21.5)	NA
≥7 years of schooling	1104 (48.2)	869 (47.7)	878 (48.4)	NA
Trade or technical qualification beyond school or university degree	342 (14.9)	256 (14.1)	260 (14.3)	NA
**Enabling resources**				
Employment status (yes), No. (%)	123 (5.4)	357 (19.7)	544 (29.9)	<0.001
Financial hardships, No. (%)				
0	1277 (58.2)	984 (54.9)	1189 (66.4)	<0.001
1–2	672 (30.6)	527 (29.4)	431 (24.1)	<0.001
≥3	245 (11.2)	280 (15.6)	171 (9.6)	0.214
Belongingness to the Australian community, No. (%)				
Hardly ever or never	177 (8.0)	66 (3.7)	98 (5.4)	<0.001
Some of the time	436 (19.6)	254 (14.1)	278 (15.4)	<0.001
Most of the time	573 (25.7)	425 (23.6)	425 (23.5)	0.088
Always	1040 (46.7)	1058 (58.7)	1008 (55.7)	<0.001
Housing arrangement, No. (%)				
Temporary	245 (10.8)	142 (7.9)	150 (8.3)	0.003
Short term lease	833 (36.7)	219 (12.2)	173 (9.5)	<0.001
Long term lease	1150 (50.7)	1321 (73.4)	1224 (67.4)	<0.001
Others	39 (1.7)	117 (6.5)	269 (14.8)	<0.001
Transport difficulty, No. (%)				
Always	453 (20.1)	174 (9.8)	185 (10.3)	<0.001
Most of the time	415 (18.4)	239 (13.5)	169 (9.4)	<0.001
Some of the time	841 (37.3)	701 (39.5)	530 (29.4)	<0.001
Never	545 (24.2)	663 (37.3)	921 (51.0)	<0.001
**Perceived needs**				
Degree of post-migration stress, mean (SD)	2.6 (2.0)	2.6 (2.0)	2.1 (1.8)	<0.001 ^2^
Overall health, mean (SD)	3.1 (1.3)	3.1 (1.4)	3.1 (1.4)	0.340 ^2^
PTSD (yes), No. (%)	735 (33.3)	581 (32.8)	513 (28.6)	0.002
HR-SMI (yes), No. (%)	378 (16.9)	346 (19.2)	306 (17.0)	0.815

Note: Numbers may not add to the column total due to missing data. ^1^ No significant difference in the frequency of MHS contacts was found between the 3rd and 5th wave based on a Kruskal–Wallis rank test. *p* values were calculated to test the unadjusted time trends and were calculated by Cochran–Armitage trend test (except ^2^ which were calculated by generalized linear mixed models for continuous variables). BNLA: Building a New Life in Australia. PTSD: post-traumatic stress disorder. HR-SMI: high risk of severe mental illness. NA: not applicable, which means that data were not measured or not reported for time-independent variables.

**Table 2:** Impact factors of having MHS contact among resettled humanitarian migrants over time.

Variables	OR (95% CI)	*p*_1_	aOR (95% CI)	*p*_2_
**Resettlement time**	1.36 (1.30, 1.42)	<0.001	1.43 (1.35, 1.52)	<0.001
**Predisposing characteristics**				
Age (year)	1.58 (1.45, 1.73)	<0.001	1.16 (1.04, 1.28)	0.006
Gender (female)	1.43 (1.20, 1.71)	<0.001	0.99 (0.82, 1.19)	0.903
Region of birth				
North Africa and the Middle East (ref)	1.00	NA	1.00	NA
South-East Asia	0.33 (0.21, 0.52)	<0.001	0.72 (0.44, 1.17)	0.185
South and Central Asia	0.82 (0.68, 1.00)	0.047	1.15 (0.91, 1,45)	0.243
Sub-Saharan Africa	1.01 (0.63, 1.63)	0.959	1.57 (0.93, 2.64)	0.090
Education level				
Never attended school (ref)	1.00	NA	1.00	NA
≤6 years of schooling	0.83 (0.62, 1.10)	0.193	1.09 (0.81, 1.48)	0.559
≥7 years of schooling	0.65 (0.50, 0.83)	0.001	0.96 (0.71, 1.28)	0.765
Trade or technical qualification beyond school or university degree	0.65 (0.47, 0.89)	0.008	0.81 (0.57, 1.17)	0.267
**Enabling resources**				
Employment status (yes)	0.39 (0.31, 0.49)	<0.001	0.71 (0.55, 0.92)	0.010
Financial hardships				
0 (ref)	1.00	NA	1.00	NA
1–2	1.47 (1.23, 1.76)	<0.001	1.04 (0.85, 1.26)	0.731
≥3	3.05 (2.42, 3.85)	<0.001	1.69 (1.31, 2.18)	<0.001
Belongingness to the Australian community				
Hardly ever or never (ref)	1.00	NA	1.00	NA
Some of the time	0.86 (0.60, 1.24)	0.425	1.19 (0.79, 1.78)	0.407
Most of the time	0.69 (0.49, 0.99)	0.044	1.14 (0.77, 1.70)	0.512
Always	0.75 (0.54, 1.06)	0.102	1.51 (1.03, 2.21)	0.034
Housing arrangement				
Temporary (ref)	1.00	NA	1.00	NA
Short term lease	0.89 (0.65, 1.22)	0.467	0.88 (0.62, 1.25)	0.468
Long term lease	0.91 (0.70, 1.20)	0.510	0.92 (0.67, 1.25)	0.579
Others	0.75 (0.51, 1.09)	0.134	0.86 (0.57, 1.32)	0.493
Transport difficulty				
Always (ref)	1.00	NA	1.00	NA
Most of the time	0.89 (0.68, 1.17)	0.412	1.10 (0.81, 1.49)	0.556
Some of the time	0.56 (0.44, 0.70)	<0.001	1.01 (0.78, 1.32)	0.931
Never	0.35 (0.28, 0.45)	<0.001	0.81 (0.61,1.08)	0.149
**Perceived needs**				
Degree of post-migration stress	1.22 (1.17, 1.27)	<0.001	1.06 (1.01, 1.11)	0.017
Overall health	1.75 (1.65, 1.86)	<0.001	1.41 (1.31, 1.52)	<0.001
PTSD (yes)	3.64 (3.09, 4.28)	<0.001	2.08 (1.72, 2.52)	<0.001
HR-SMI (yes)	4.71 (3.92, 5.67)	<0.001	1.99 (1.59, 2.49)	<0.001

Note: MHS: mental health service; OR: odds ratio, which was calculated by univariate GLMMs; aOR: adjusted odds ratio, which was calculated by the multivariable GLMM; CI: confidence interval; ref: reference group; NA: not applicable, which means *p* values are not applicable for the reference group; PTSD: post-traumatic stress disorder; HR-SMI: high risk of severe mental illness.

**Table 3:** Factors associated with the frequency of MHS contacts among resettled humanitarian migrants.

Variables	OR (95% CI)	*p*_1_	aOR (95% CI)	*p*_2_
**Predisposing characteristics**				
Age (year)	1.02 (1.01, 1.04)	<0.001	1.00 (0.99, 1.02)	0.625
Gender (female)	1.57 (1.14, 2.15)	0.005	1.50 (1.04, 2.16)	0.032
Education level				
Never attended school (ref)	1.00	NA	1.00	NA
≤6 years of schooling	0.76 (0.52, 1.11)	0.151	0.90 (0.59, 1.40)	0.651
≥7 years of schooling	1.67 (1.18, 2.35)	0.003	1.54 (1.05, 2.26)	0.029
Trade or technical qualification beyond school or university degree	1.04 (0.77, 1.40)	0.790	1.07 (0.77, 1.48)	0.689
**Enabling resources**				
Employment status (yes)	0.40 (0.26, 0.61)	<0.001	0.71 (0.43, 1.18)	0.191
Financial hardships				
0 (ref)	1.00	NA	1.00	NA
1–2	1.70 (1.18, 2.46)	0.005	1.28 (0.84, 1.96)	0.257
≥3	1.49 (0.90, 2.45)	0.119	1.24 (0.70, 2.20)	0.457
Belongingness to the Australian community				
Hardly ever or never (ref)	1.00	NA	1.00	NA
Some of the time	1.16 (0.55, 2.41)	0.700	1.22 (0.53, 2.80)	0.637
Most of the time	1.56 (0.76, 3.18)	0.225	1.63 (0.72, 3.70)	0.240
Always	1.00 (0.52, 1.94)	0.994	1.30 (0.60, 2.80)	0.508
**Perceived needs**				
Degree of post-migration stress	1.12 (1.02, 1.22)	0.014	1.03 (0.93, 1.15)	0.578
Overall health	1.62 (1.43, 1.84)	<0.001	1.45 (1.22, 1.71)	<0.001
PTSD (yes)	2.66 (1.92, 3.69)	<0.001	1.93 (1.33, 2.82)	<0.001
HR-SMI (yes)	2.54 (1.79, 3.59)	<0.001	1.44 (0.94, 2.19)	0.094

Note: MHS: mental health services; OR: odds ratio, which was calculated by univariate ordered logistic regression models; aOR: adjusted odds ratio, which was calculated by the multivariable ordered logistic regression models; ref: reference group; NA: not applicable, which means *p* values are not applicable for the reference group; PTSD: post-traumatic stress disorder; HR-SMI: high risk of severe mental illness.

## Data Availability

The BNLA dataset was publicly archived and available for authorized people at the National Centre for Longitudinal Data website (https://www.dss.gov.au/national-centrefor-longitudinal-data-ncld/access-to-dss-longitudinal-datasets (accessed on 18 November 2020)).

## Supplementary Materials

The following supporting information can be downloaded at: https://www.mdpi.com/article/10.3390/ijerph191610119/s1, Figure S1: Conceptual framework adapted from Andersen’s framework to explore impact factors of MHS utilization; Table S1: Definitions of study variables; Table S2: The frequency of MHS contacts among resettled humanitarian migrants at the 5th wave; Table S3: Trends of the prevalence of common mental health conditions among resettled humanitarian migrants; Table S4: Sensitivity analysis of impact factors of having MHS contacts among resettled humanitarian migrants over time; Table S5: Sensitivity analysis of factors associated with the frequency of MHS contacts among humanitarian migrants.
