# Supplementary material for: Trends and Impact Factors of Mental Health Service Utilization among Resettled Humanitarian Migrants in Australia: Findings from the BNLA Cohort Study
Source: Int J Environ Res Public Health. 2022 Aug 16;19(16):10119. doi: 10.3390/ijerph191610119 (PMC9408151; doi:10.3390/ijerph191610119)
Supplement: Supplementary file 1 [file ijerph-19-10119-s001.zip › ijerph-1809379-supplementary.pdf]

Supplementary Materials

Trends and impact factors of mental health service utilization among resettled humanitarian migrants in Australia: findings from the BNLA cohort study

Meng Zheng [1,2], Feng Chen [1,2], Yan Pan [1,2], Di Kong [1,2], Andre M.N. Renzaho [3,4], Berhe W. Sahle [5,6,7], Rashidul Alam Mahumud [8,9], Li Ling [1,2] and Wen Chen [1,2,*]

contents

Figure S1. Conceptual framework adapted from Andersen's framework to explore impact factors of MHS utilization 2

Table S1. Definitions of study variables 3

Table S2. The frequency of MHS contacts among resettled humanitarian migrants at the 5th wave 5

Table S3. Trends of the prevalence of common mental health conditions among resettled humanitarian migrants 6

Table S4. Sensitivity analysis of impact factors of having MHS contacts among resettled humanitarian migrants over time 7

Table S5. Sensitivity analysis of factors associated with the frequency of MHS contacts among humanitarian migrants 8

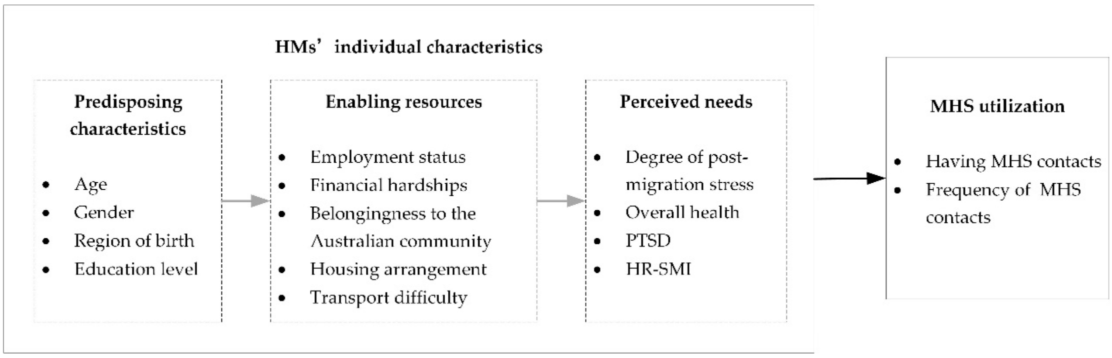

Figure S1. Conceptual framework adapted from Andersen's framework to explore impact factors of MHS utilization. Note: PTSD: post-traumatic stress disorder; HR-SMI: high risk of severe mental illness; MHS: mental health services.

Table S1. Definitions of study variables.

	Questions	Response options and coding
**MHS utilization**		
Having MHS contacts	Have you been given any medication by a doctor for emotional problems since you arrived in Australia? Have you received help for emotional problems in the last 12 months?	The response was dichotomized (1 = "yes" if the participants reported MHS utilization in the duration or 0 = "no" otherwise).
Frequency of MHS contacts	How often have you received help for emotional problems in the last 12 months?	The responses were coded as "1 = 1-2 times", "2 = 3-5 times", "3 = 6-9 times", "4 = ≥10 times".
**Predisposing characteristics**		
Age	How old are you?	Responses were numbers of years
gender	What's your sex?	1= male, 2= female
Region of birth	You were born in…?	Responses were recorded as 1 = North Africa and the Middle East; 4 = South-East Asia; 7 = South and Central Asia; 9 = Sub-Saharan Africa
Education level	What is the highest level of education you completed before coming to Australia?	Education levels were categorized as follows: never attended school, ≤6 years of schooling, ≥7 years of schooling, trade or technical qualification beyond school or university degree
**Enabling resources**		
Employment status	In the last 7 days did you do any paid work in a job?	The response was dichotomized (1 = "yes" or 0 = "no" otherwise).
Financial hardships	Has any of the following happened to you because you didn't have enough money: 1) Couldn't pay gas, electricity, or telephone bills on time? 2) Couldn't pay the rent or mortgage payments on time? 3) Went without meals? 4) Were unable to heat or cool your home? 5) Pawned or sold something because you need crash? 6) Needed help from a welfare or community organization?	0 = no financial hardship, to 6 = 6 kinds of financial hardships. According to existing literature, the numbers were further categorized as follows: 0 = no financial hardships, 1 = 1 to 2 financial hardships, 3 = 3 or more financial hardships
belongingness to the Australian community	Do you feel part of the Australian community?	The responses were recorded as never or hardly ever, some of the time, most of the time, always (strong)
Housing arrangement	Is your current housing arrangement…	The responses were coded as temporary, short term lease, long term lease, others
Transport difficulty	How often do you have difficulty traveling to the places you need to go?	The responses were coded as always, most of the time, some of the time, never
**Perceived needs**		

	Questions	Response options and coding
Degree of post-migration stress	Are any of the following a source of stress in your life? 1) work situation; 2) house situation; 3) financial situation; 4) loneliness; 5) language barriers; 6) discrimination; 7) getting used to life in Australia; 8) caring for family health; 9) family's safety; 10) conflict/tension with partner	0 = having no life stressor, to 10 = having 10 kinds of life stressors.
Overall health	Overall, how would you rate your health during the past 4 weeks?	Responses: excellent, very good, good, fair, poor, very poor.
PTSD	PTSD-8 (8 items): Intrusion (recurrent thoughts or memories of the events, feeling as though the event is happening again, recurrent nightmares, sudden emotional or physical reaction when remind of the events), Avoidance (avoiding activities that remind you of the events, avoiding thoughts or feelings associated with the events), Hypervigilance (feeling jumpy or easily startled, feeling on guard)	For each item, responses were not at all, rarely, sometimes, or most of the time. The prevalence of PTSD was defined as dichotomized (1= "yes" if at least one symptom from each of the three PTSD-8 subscales has an item with an answer "sometimes" or "most of the time".
HR-SMI	K6 (6 items: nervous, hopeless, restless and fidgety, everything an effort, nothing cheer up, worthless)	For each item, the response was 1= none of the time, to 5= all of the time, and a total score of 6-30. Responses were recorded as dichotomized (1= "yes" if the total score was 19 or more, 0= "no" otherwise).

Note: MHS: mental health service; PTSD: post-traumatic stress disorder; HR-SMI: high risk of severe mental illness.

Table S2. The frequency of MHS contacts among resettled humanitarian migrants at the 5th wave.

	the frequency of MHS contacts in the past 12 months				
	**1-2 times (n = 119)**	**3-5 times (n = 157)**	**6-9 times (n = 82)**	**≥10 times (n = 141)**	***p***
Predisposing characteristics					
Age, mean(SD), year	37.3 (13.3)	39.8 (14.6)	45.1 (12.4)	42.9 (12.1)	<0.001
Gender (female), No.(%)	54 (45.4)	71 (45.2)	40 (48.8)	87 (61.7)	0.005
Region of birth, No.(%)					0.941
North Africa and the Middle East	81 (68.1)	103 (65.6)	52 (63.4)	96 (68.1)	
South-East Asia	1 (0.8)	3 (1.9)	1 (1.2)	0	
South and Central Asia	35 (29.4)	47 (29.9)	27 (32.9)	42 (29.8)	
Sub-Saharan Africa	2 (1.7)	4 (2.6)	2 (2.4)	3 (2.1)	
Education level, No.(%)					0.019
Never attended school	16 (13.5)	20 (12.9)	22 (26.8)	35 (25.2)	
≤6 years of schooling	28 (23.5)	44 (28.4)	19 (23.2)	31 (22.3)	
≥7 years of schooling	61 (51.3)	70 (45.2)	25 (30.5)	53 (85.6)	
Trade or technical qualification beyond school or university degree	14 (11.8)	21 (13.6)	16 (19.5)	20 (14.4)	
Enabling resources					
Employment status (yes), No.(%)	32 (27.4)	34 (21.8)	11 (13.6)	11 (8.0)	<0.001
Financial hardships, No.(%)					0.050
0	79 (67.0)	94 (61.8)	50 (62.5)	68 (48.6)	
1-2	26 (22.0)	37 (24.3)	24 (30.0)	49 (35.0)	
≥3	13 (11.0)	21 (13.8)	6 (7.5)	23 (16.4)	
Belongingness to the Australian community, No.(%)					0.046
Hardly ever or never	11 (9.3)	9 (5.8)	4 (5.1)	11 (8.0)	
Some of the time	26 (22.0)	19 (12.3)	15 (19.0)	28 (20.3)	
Most of the time	22 (18.6)	31 (20.1)	14 (17.7)	41 (29.7)	
Always	59 (50.0)	95 (61.7)	46 (58.2)	58 (42.0)	
Housing arrangement, No.(%)					0.152
Temporary	13 (11.0)	10 (6.4)	5 (6.1)	8 (5.7)	
Short term lease	16 (13.6)	9 (5.7)	10 (12.2)	16 (11.4)	
Long term lease	75 (63.6)	111 (70.7)	58 (70.7)	104 (74.3)	
Other	14 (11.8)	27 (17.2)	9 (11.0)	12 (8.6)	
Transport difficulty, No.(%)					0.091
Always	20 (17.1)	23 (14.7)	13 (16.1)	36 (25.5)	
Most of the time	14 (12.0)	16 (10.2)	8 (9.9)	25 (17.7)	
Some of the time	41 (35.0)	50 (31.9)	28 (34.6)	35 (24.8)	
Never	42 (35.9)	68 (43.3)	32 (39.5)	45 (31.9)	
Perceived needs					
Degree of post-migration stress, mean(SD)	2.3 (2.0)	2.4 (1.9)	2.5 (1.7)	2.9 (1.9)	0.024
Overall health, mean(SD)	3.5 (1.3)	3.8 (1.3)	4.1 (1.2)	4.7 (1.1)	<0.001
PTSD (yes), No.(%)	41 (35.0)	67 (43.5)	39 (48.2)	99 (71.2)	<0.001
HR-SMI (yes), No.(%)	28 (23.5)	42 (27.3)	25 (31.3)	75 (53.6)	<0.001

Note: Numbers may not add to the column total due to missing data. MHS: mental health services; PTSD: post-traumatic stress disorder; HR-SMI: high risk of severe mental illness; p values were calculated by chi-square test for categorical variables, and by Kruskal-Wallis rank-sum test for continuous variables, variables with $p \leq 0.05$ were included in the multivariable ordered logistic model.

Table S3. Trends of the prevalence of common mental health conditions among resettled humanitarian migrants.

	Wave 1				Wave 3				Wave 5			
	**Total**	**Females**	**Males**	p_1	**Total**	**Females**	**Males**	p_3	**Total**	**Females**	**Males**	p_5
PTSD	735/2205 (33.3%)	383/1014 (37.8%)	352/1191 (29.6%)	<0.001	581/1773 (32.8%)	312/841 (37.1%)	269/932 (28.9%)	<0.001	513/1794 (28.6%)	274/852 (32.7%)	239/942 (25.4%)	0.001
HR-SMI	378/2239 (16.9%)	230/1034 (22.2%)	148/1205 (12.3%)	<0.001	346/1799 (19.2%)	204/848 (24.1%)	142/951 (14.9%)	<0.001	306/1799 (17.0%)	166/857 (19.4%)	140/942 (14.9%)	0.011
PTSD and/or HR-SMI	837/2194 (38.1%)	447/1013 (44.1%)	390/1181 (33.0%)	<0.001	690/1765 (39.1%)	374/835 (44.8%)	316/930 (34.0%)	<0.001	600/1789 (33.5%)	320/852 (37.6%)	280/937 (29.9%)	0.001

Note: Data are n/N (%), where n refers to the number of participants with the corresponding mental health conditions, and N refers to the number of participants who reported the data. Especially, for data of PTSD and/or HR-SMI, N means the number of participants who reported both data of PTSD and HR-SMI, and the participants who reported having one of the mental health conditions with a missing value in another. p values were calculated by chi-square tests for the gender differences in the prevalence of common health conditions, with all $p < 0.05$. PTSD: post-traumatic stress disorder; HR-SMI: high risk of severe mental illness.

Table S4. Sensitivity analysis of impact factors of having MHS contacts among resettled humanitarian migrants.

	Unweighted data		Weighted data	
	**aOR (95% CI)**	***p***	**aOR (95% CI)**	***p***
Resettlement time	1.43 (1.35, 1.52)	<0.001	1.35 (1.28, 1.43)	<0.001
Predisposing characteristics				
Age	1.16 (1.04, 1.28)	0.006	1.14 (1.04, 1.26)	0.008
Gender (female)	0.99 (0.82, 1.19)	0.903	1.02 (0.85, 1.23)	0.801
Region of birth				
North Africa and the Middle East (ref)	1.00	NA	1.00	NA
South-East Asia	0.72 (0.44, 1.17)	0.185	0.68 (0.46, 1.02)	0.061
South and Central Asia	1.15 (0.91, 1,45)	0.243	1.09 (0.86, 1.36)	0.479
Sub-Saharan Africa	1.57 (0.93, 2.64)	0.090	1.35 (0.87, 2.09)	0.175
Education level				
Never attended school (ref)	1.00	NA	1.00	NA
≤6 years of schooling	1.09 (0.81, 1.48)	0.559	1.08 (0.80, 1.45)	0.636
≥7 years of schooling	0.96 (0.71, 1.28)	0.765	0.92 (0.69, 1.22)	0.561
Trade or technical qualification beyond school or university degree	0.81 (0.57, 1.17)	0.267	0.79 (0.56, 1.13)	0.194
Enabling resources				
Employment status (yes)	0.71 (0.55, 0.92)	0.010	0.74 (0.57, 0.96)	0.022
Financial hardships				
0 (ref)	1.00	NA	1.00	NA
1-2	1.04 (0.85, 1.26)	0.731	1.08 (0.88, 1.32)	0.464
≥3	1.69 (1.31, 2.18)	<0.001	1.86 (1.44, 2.40)	<0.001
Belongingness to the Australian community				
Hardly ever or never (ref)	1.00	NA	1.00	NA
Some of the time	1.19 (0.79, 1.78)	0.407	1.10 (0.73, 1.65)	0.641
Most of the time	1.14 (0.77, 1.70)	0.512	1.04 (0.70, 1.55)	0.828
Always	1.51 (1.03, 2.21)	0.034	1.40 (0.96, 2.05)	0.083
Housing arrangement				
Temporary (ref)	1.00	NA	1.00	NA
Short term lease	0.88 (0.62, 1.25)	0.468	0.76 (0.53, 1.07)	0.113
Long term lease	0.92 (0.67, 1.25)	0.579	0.79 (0.58, 1.07)	0.125
Others	0.86 (0.57, 1.32)	0.493	0.78 (0.52, 1.18)	0.242
Transport difficulty				
Always (ref)	1.00	NA	1.00	NA
Most of the time	1.10 (0.81, 1.49)	0.556	1.07 (0.79, 1.46)	0.657
Some of the time	1.01 (0.78, 1.32)	0.931	1.09 (0.83, 1.42)	0.550
Never	0.81 (0.61, 1.08)	0.149	0.85 (0.64, 1.13)	0.272
Perceived needs				
Degree of post-migration stress	1.06 (1.01, 1.11)	0.017	1.07 (1.02, 1.12)	0.006
Overall health	1.41 (1.31, 1.52)	<0.001	1.36 (1.26, 1.47)	<0.001
PTSD (yes)	2.08 (1.72, 2.52)	<0.001	2.14 (1.76, 2.59)	<0.001
HR-SMI (yes)	1.99 (1.59, 2.49)	<0.001	1.98 (1.57, 2.49)	<0.001

Note: GLMMs: generalized linear mixed models; ref: reference group; NA: not applicable, which means *p* values are not applicable for the reference group; PTSD: post-traumatic stress disorder; HR-SMI: high risk of severe mental illness.

Table S5. Sensitivity analysis of factors associated with the frequency of MHS contacts among humanitarian migrants.

	Complete data		Imputed data	
	**aOR (95% CI)**	***p***	**aOR (95% CI)**	***p***
Predisposing characteristics				
Age	1.00 (0.99, 1.02)	0.625	1.00 (0.99, 1.02)	0.644
Gender (female)	1.50 (1.04, 2.16)	0.032	1.39 (0.98, 1.96)	0.062
Education level				
Never attended school (ref)	1.00	NA	1.00	NA
≤6 years of schooling	0.90 (0.59, 1.40)	0.648	0.92 (0.61, 1.38)	0.682
≥7 years of schooling	1.54 (1.05, 2.26)	0.029	1.56 (1.08, 2.27)	0.018
Trade or technical qualification beyond school or university degree	1.07 (0.77, 1.48)	0.689	1.09 (0.80, 1.49)	0.589
Enabling resources				
Employment status (yes)	0.71 (0.43, 1.18)	0.191	0.62 (0.39, 1.00)	0.050
Financial hardships				
0 (ref)	1.00	NA	1.00	NA
1-2	1.28 (0.84, 1.96)	0.257	1.26 (0.84, 1.89)	0.266
≥3	1.24 (0.70, 2.20)	0.457	1.12 (0.65, 1.91)	0.682
Belongingness to the Australian community				
Hardly ever or never (ref)	1.00	NA	1.00	NA
Some of the time	1.22 (0.53, 2.80)	0.637	1.31 (0.60, 2.84)	0.499
Most of the time	1.63 (0.72, 3.70)	0.240	1.87 (0.87, 4.02)	0.110
Always	1.30 (0.60, 2.80)	0.508	1.49 (0.74, 3.03)	0.266
Perceived needs				
Degree of post-migration stress	1.03 (0.93, 1.15)	0.578	1.05 (0.95, 1.16)	0.340
Overall health	1.45 (1.22, 1.71)	<0.001	1.38 (1.18, 1.60)	<0.001
PTSD (yes)	1.93 (1.33, 2.82)	<0.001	1.90 (1.33, 2.72)	<0.001
HR-SMI (yes)	1.44 (0.94, 2.19)	0.094	1.41 (0.95, 2.10)	0.090

Note: In 499 participants who reported the frequency of contacts, 445 humanitarian migrants who had no missing data were included in the sample of complete data, another 54 (10.8%) participants have missing data in independent variables. The highest missing rate across independent variables was 3.8%, which occurred in the "degree of post-migration stress". Missing data were imputed by the multiple imputation method. MHS: mental health service; ref: reference group; NA: not applicable, which means that *p* is not calculates for the reference group; PTSD: post-traumatic stress disorder; HR-SMI: high risk of severe mental illness.
